# Highly Thermo-Conductive Three-Dimensional Graphene Aqueous Medium

**DOI:** 10.1007/s40820-020-00478-2

**Published:** 2020-07-01

**Authors:** Zheng Bo, Chongyan Ying, Huachao Yang, Shenghao Wu, Jinyuan Yang, Jing Kong, Shiling Yang, Yanguang Zhou, Jianhua Yan, Kefa Cen

**Affiliations:** 1 State Key Laboratory of Clean Energy Utilization, Institute for Thermal Power Engineering, College of Energy Engineering, Zhejiang University, 38 Zheda Road, Hangzhou, 310027 People’s Republic of China; 2 ZJU-Hangzhou Global Scientific and Technological Innovation Center, Hangzhou, 311215 People’s Republic of China; 3 Department of Mechanical and Aerospace Engineering, The Hong Kong University of Science and Technology, Clear Water Bay, Kowloon, Hong Kong People’s Republic of China

**Keywords:** Three-dimensional graphene, Thermo-conductive aqueous medium, Multiscale modeling, Solar thermal conversion, Practical thermal management

## Abstract

Highly thermo-conductive aqueous medium is a crucial premise to demonstrate high-performance thermal-related applications. Graphene has the diamond comparable thermal conductivity, while the intrinsic two-dimensional reality will result in strong anisotropic thermal conductivity and wrinkles or even crumples that significantly sacrifices its inherent properties in practical applications. One strategy to overcome this is to use three-dimensional (3D) architecture of graphene. Herein, 3D graphene structure with covalent-bonding nanofins (3D-GS-CBF) is proposed, which is then used as the filler to demonstrate effective aqueous medium. The thermal conductivity and thermal conductivity enhancement efficiency of 3D-GS-CBF (0.26 vol%) aqueous medium can be as high as 2.61 W m^−1^ K^−1^ and 1300%, respectively, around six times larger than highest value of the existed aqueous mediums. Meanwhile, 3D-GS-CBF can be stable in the solution even after 6 months, addressing the instability issues of conventional graphene networks. A multiscale modeling including non-equilibrium molecular dynamics simulations and heat conduction model is applied to interpret experimental results. 3D-GS-CBF aqueous medium can largely improve the solar vapor evaporation rate (by 1.5 times) that are even comparable to the interfacial heating system; meanwhile, its cooling performance is also superior to commercial coolant in thermal management applications.

**Electronic supplementary material:**

The online version of this article (10.1007/s40820-020-00478-2) contains supplementary material, which is available to authorized users.

## Highlights

- 3D graphene structure with covalent-bonding nanofins is proposed to demonstrate highly thermo-conductive aqueous medium.
- An ultralow loading of 3D graphene enables aqueous medium with a record high thermal conductivity of 2.61 W m^−1^ K^−1^.
- 3D graphene aqueous medium can remarkably enhance the performance of solar thermal conversion and heat dissipation.

**Electronic supplementary material:**

The online version of this article (10.1007/s40820-020-00478-2) contains supplementary material, which is available to authorized users.

## Graphical abstract

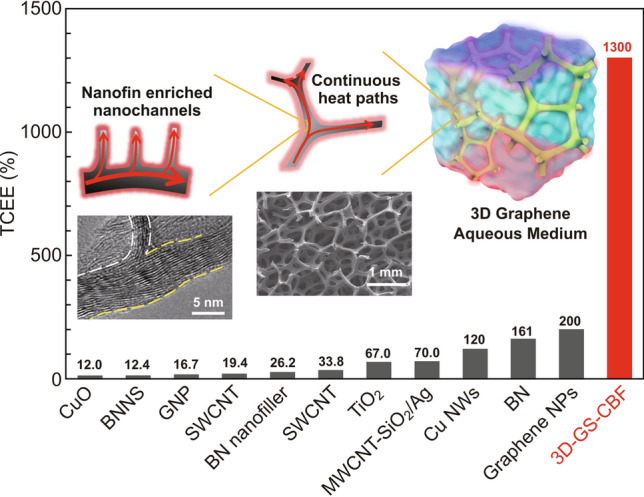

## Introduction

The excellent thermal transport property of graphene (3000–5000 W m^−1^ K^−1^) comparable to diamond [1–7], makes it as a potential candidate for thermal energy dissipation materials. Unlike the traditional three-dimensional (3D) materials such as diamond, the intrinsic two-dimensional (2D) reality of graphene will result in strong anisotropic thermal conductivity and wrinkles or even crumples [8, 9] that significantly sacrifices its inherent properties in practical applications. For example, the single or multilayer graphenes are easily folded or curved in the aqueous medium and cannot maintain its 2D form to achieve superior in-plane thermal conductivity. One strategy to solve this issue is to use the graphene together with the substrate. However, the structures as well as the properties of substrate affect the thermal transport properties strongly. For example, the micro-Raman spectroscopy for suspended graphene reveals an extremely high thermal conductivity of 2000–5000 W m^−1^ K^−1^ [7, 10], while a significantly reduced value of ~ 600 W m^−1^ K^−1^ is observed for exfoliated graphene on SiO_2_/Si substrate [4]. The reduced experimental values have been attributed to the graphene-substrate coupling and phonon leaking across the interface. Another strategy is to fabricate freestanding 3D graphene architectures. It has been proved that the 3D graphene structures may have excellent mechanical [11–15], electrical [14–17] and thermal properties [18–20]. For the thermal applications, graphene is often applied to be as the filler in the aqueous medium which can be used for solar thermal conversion [21, 22], photothermal catalysis [22] and chip’s cooling process [23], etc. In these thermal applications, the thermal transport between water and filler which determines the global thermal conductivity of the aqueous medium is the key role in determining the bulk heating rate and related application performance [24]. For instance, the rapid heat exchange between fillers and the surrounding water can increase the global heating and temperature rise of the aqueous medium and therefore enlarge solar vapor evaporation rate [21].

**Fig. 1:**
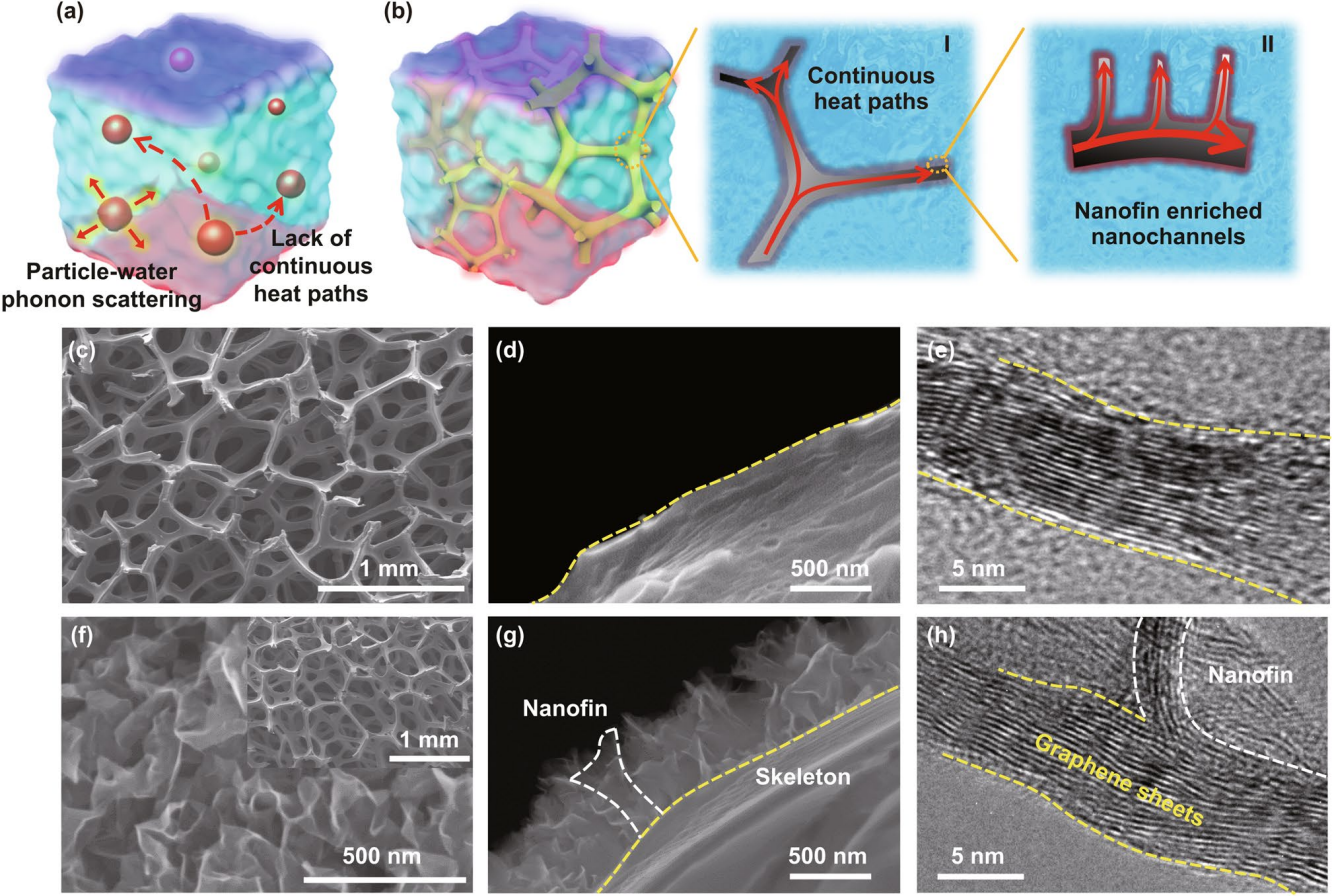
**a**, **b** Schematic illustration of conventional aqueous medium and our 3D graphene system. **c–e** Top- and cross-view SEM images and TEM image of the skeleton of 3D-GS sample. **f–g** Top- and cross-view SEM images of graphene nanofins on the skeleton surface of 3D-GS-CBF sample. The inset in **f** denotes the SEM image of 3D-GS-CBF. **h** HRTEM image of the conjunction of nanofin and skeleton

In order to improve the thermal performance of the aqueous medium, a popular strategy is to add high thermally conductive nanofillers such as graphene-related materials and structures [25–28]. The super-high thermal conductivity of graphene has offered a window for enhancing the thermal transport properties of the aqueous medium largely [28–30]. In general, with a low graphene loading of < 5.0 vol%, the thermal conductivity of aqueous medium can be improved to 1.0 W m^−1^ K^−1^ [28–31], while this is still far behind the theoretical expectations. To reach the superior thermal conductivity (> 2.0 W m^−1^ K^−1^), an ultra-large amount of nanofiller (typically > 20.0 vol%) is inevitably required which may cause high costs, heavy weight and agglomeration issues [30]. The reason for the poor performance of the aqueous medium is because of the coarsely designed structures and the discrete filler distribution in the solutions (Fig. 1a), in which a large filler loading up to > 30 vol% is typically required to form continuous thermal transport channels. Meanwhile, the monolayer or multilayer graphenes are easily folded or curved in the aqueous medium and cannot maintain its 2D form to achieve inherent high thermal conductivity, leading to a relatively low thermal conductivity enhancement efficiency (TCEE) of < 200% [32]. 3D graphene networks, assembled via non-covalent interactions through freeze casting and chemical methods, etc., exhibit great potential to improve the performance of the aqueous medium [19], while the inherent instability issues of this kind structure in aqueous medium, e.g., quite easily disperse and reaggregate into the solutions [33, 34], have hindered its practical long-term applications.

In this paper, we suggest an efficient aqueous medium based on the 3D graphene continuous structure with covalent-bonding nanofins (3D-GS-CBF) on the skeleton. In the 3D-GS-CBF, apart from the graphene skeleton which conducts heat, the nanofins covalently grown on the top of the skeleton can exchange energy with the liquid medium as well. As a result, the heat transfer at the graphene–liquid medium interfaces can be improved largely (Fig. 1b). With a quite small loading of 0.26 vol% 3D-GS-CBF, the thermal conductivity of 3D-GS-CBF aqueous medium can be as high as ~ 2.61 W m^−1^ K^−1^, which yields a record high TCEE of ~ 1300%. Meanwhile, it exhibits a long-term stability (> 6 months) in the solutions, resolving the instability issues of conventional non-covalent graphene structures. We further implement multiscale modeling technique (including non-equilibrium molecular dynamics simulations and heat conduction equations) to study the underlying mechanisms. Two examples of the solar thermal conversion and LED thermal management processes are then used to prove the potential thermal application of the 3D-GS-Fin aqueous medium.

## Methods

### Fabrication and Characterization of the Graphene-Based Structures

#### Material Fabrication

The commercial Ni foam (1.6 mm in thickness) was cut into pieces with the lateral size needed. Then, it was placed into a cylindrical quartz tube (internal diameter: 43 mm). The quartz tube was first evacuated to a low pressure of 3 Pa and then heated up to 700 °C. An inductively coupled plasma (ICP) source with a radiofrequency power of 250 W was employed. A mixture of CH_4_ (5 mL min^−1^) and H_2_ (5 mL min^−1^) was introduced into the quartz tube. The pressure of chamber was maintained at 30 Pa during the growth procedure. After deposition for 1 h, the system was cooled to room temperature. The as-synthesized graphene-Ni foam was immersed in polymethyl methacrylate (PMMA) solution (4 wt% in ethyl lactate) for seconds and then dried at 80 °C for 2 h. The PMMA-coated graphene-Ni foam was immersed into a 3 M HCl solution at 80 °C overnight to dissolve the Ni template. After removing the PMMA with hot acetone solution at 50 °C, the hierarchical and free-standing 3D-GS-CBF was obtained. The dried sample was then functional group modified by 500 ppm moist ozone flow (1 L min^−1^) for 5 min. As for the preparation of 3D-GS, the commercial Ni foam with the same size was heated to 1000 °C in a quartz tube under a mixture of Ar (500 mL min^−1^) and H_2_ (200 mL min^−1^) for 5 min. Thereafter, CH_4_ of 10 mL min^−1^ was introduced into the reaction tube at ambient pressure. After feeding the reaction-gas mixture flow for 5 min, the sample was rapidly cooled to room temperature at a cooling rate of ∼100 °C min^−1^ under the mixed H_2_ (200 mL min^−1^) and Ar (500 mL min^−1^) flow. The etching process was consistent with those described in the previous work.

#### Material Characterization

The morphology and structure of sample were inspected by scanning electron microscope (SEM, Hitachi SU-70) and transmission electron microscope (TEM, Tecnai G2 F20S-TWIN). Raman spectra were confirmed by Raman spectrometer (LabRAM HR Evolution) with a 532-nm laser beam. X-ray photoelectron spectroscopy (XPS) measurement was taken through VG ESCALAB MARK II spectrometer. The contact angles of samples were tested by a computerized contact angle analyzer (DropMeter A-200, MAIST), and images of droplet impact were captured with high-speed camera (REDLAKE MotionXtra HG-100 K). The thermal diffusivity *α* of aqueous medium was measured by a laser flash analysis apparatus (NETZSCH LFA467 Nanoflash). Then the thermal conductivity *k* could be calculated as *k* = *ραc*. The thermal conductivity enhancement (TCE) is defined as TCE = (*k *−*k*_water_)/*k*_water_ × 100%, where *k*_water_ denotes thermal conductivity of water. The thermal conductivity enhancement efficiency (TCEE) is defined as TCEE = TCE*/ϕ*, where *ϕ* presents the fraction of filler in water.

### Measurement of Heat Exchange Process

#### Experimental Setup of Solar Thermal Conversion Application

Simulated solar light was generated by a xenon lamp (PLS-SXE300D). The light intensity at test spot was kept at 1000 W/m^2^, which was measured by light intensity meter (PD130). A quartz container with an inner diameter of 26 and 6 mm in depth was put at the solar thermal position. A thermocouple (TT-T-36) was fixed at the bottom of the container to measure the temperature change, and an electric balance (CPA225D, Sartorius) was used to record the mass change for calculating the evaporation rate. The solar vapor generation rate can be calculated as:

1 $$\mathop m\limits^{ \bullet } = \frac{{{{\Delta m} \mathord{\left/ {\vphantom {{\Delta m} {\Delta t}}} \right. \kern-\nulldelimiterspace} {\Delta t}}}}{A}$$

where $\mathop m\limits^{ \bullet }$ is the steady-state evaporation rate (kg m^−2^ h^−1^), *m* and *t* are the evaporation amount of the stabilization state, respectively, *A* is the area of evaporation setup. Afterward, solar vapor generation efficiency can be defined as:

2 $$\eta = \frac{{\left( {\mathop m\limits^{ \bullet } - \mathop {m_{0} }\limits^{ \bullet } } \right)h_{{{\text{lv}}}} }}{I}$$

where $\mathop {m_{0} }\limits^{ \bullet }$ denotes the evaporation rate in dark environment, $h_{{{\text{lv}}}}$ is the latent heat of phase change of water, and *I* is the power density of the light (W m^−2^).

#### Experimental Setup of LED Thermal Management Applications

The experimental setup consisted of a pump, a tank and a DC power supply (UTP1306S). The test section consisted of a water block, a high-power LED light and thermocouples. The water block was assembled by a poly-(methyl methacrylate) (PMMA) cover and an aluminum plate with an interspace of 40 × 40 × 2.5 mm. High-power LED light (32 V, 20 W) was attached on Al plate by a layer of thermal interfacial material. Three thermocouples were applied to measure the temperature of the LEDs, inlet, and outlet water, respectively. The operating current of LEDs was recorded using a Keithley 2602 source meter.

### Heat Exchange Process Simulations

#### Non-equilibrium Molecular Dynamics (NEMD) Simulations

The inter-atomic interactions of carbon atoms in graphene sheets were characterized by the adaptive intermolecular reactive empirical bond order (AIREBO) potential, which can accurately describe the carbon nanostructure system. The REBO function is defined mathematically as:

3 $$V_{REBO} = V_{R} \left( {r_{ij} } \right) - b_{ij} V_{A} \left( {r_{ij} } \right)$$

where *V*_*A*_ and *V*_*R*_ denote the attractive and repulsive pair terms, respectively; *b*_*ij*_ term presents the reactive empirical bond order between carbon atoms. In the extension from REBO to AIREBO potential, non-bonded interactions and dihedral-angle interactions term are added, i.e.,

4 $$V_{kijl}^{{{\text{AIREBO}}}} = \frac{1}{2}\sum\limits_{i} {\sum\limits_{i \ne j} {\left[ {V_{ij}^{{{\text{REBO}}}} + V_{ij}^{LJ} + \sum\limits_{k \ne i} {\sum\limits_{jl \ne i,j,k} {V_{kijl}^{{{\text{tors}}}} } } } \right]} }$$

where $V_{ij}^{{{\text{LJ}}}}$ and $V_{kijl}^{{{\text{tors}}}}$ present the L-J potential and dihedral-angle term, respectively.

Water molecules were described by the simple point charge extended model, and Shake algorithm was used to constrain the bond and angle of water molecules. Lennard–Jones (LJ) potentials were employed to characterize the interactions between graphene and water molecules. Based on the XPS results, hydroxyl groups were also applied on the graphene surface to accurately describe the interfacial interactions. In these models, the interactions between atomic sites can be expressed as:

5 $$E_{ij} = \frac{{q_{i} q_{j} }}{{r_{ij} }} + 4\varepsilon \left[ {\left( {\frac{{\sigma_{ij} }}{{r_{ij} }}} \right)^{12} - \left( {\frac{{\sigma_{ij} }}{{r_{ij} }}} \right)^{6} } \right]$$

where *q*_*i*_, *r*_*ij*_, *ε*_*i*_ and *σ*_*i*_ represent the charge of *i*_th_ atom, the distance between *i*_th_ and *j*_th_ atom, the minimum energy and zero energy separation distance, respectively. The inter-atomic LJ parameters between different species were calculated based on Lorentz–Berthelot mixing rules (Table 1).

**Table 1:** LJ parameters and partial charges for graphene, water molecules and functional groups

Parameters	Atom	*Σ* (Å)	*ε* (kJ mol^−1^)	*q* (*e*)
Graphene	C	3.400	0.2330	0.000
Water	OW	3.166	0.6502	−0.8476
	HW	0.0000	0.0000	0.4238
OH	C(graphene)	3.400	0.2330	0.265
	O	3.070	0.7117	−0.700
	H	0.000	0.000	0.435

The non-equilibrium molecular dynamics (NEMD) simulations were performed by Large-scale Atomic/Molecular Massively Parallel Simulator (LAMMPS) [35]. A cutoff of 12.0 Å was used for calculating the van der Waals and Coulombic interactions in the real space, and the long-range Coulombic interactions were treated by particle–particle particle-mesh (PPPM) algorithm (root-mean-square accuracy of 10^–6^). Periodic boundary conditions are applied in all directions. During the MD simulation, the system is firstly relaxed for 5 ns in the NVT ensemble, followed by NPT ensemble for 2 ns (at 1 atm and 300 K). Subsequently, an extra 2 ns in NVE ensemble is carried out to confirm the equilibrium state of system. To get steady temperature gradient, a heat source (350 K) and a heat sink (250 K) are set on the left and right graphene atoms, respectively. When the steady temperature profiles are established, the interfacial thermal resistance can be calculated as *R*_*b*_ = *AΔT*/*J*, where *J* is the heat flux, *ΔT* is the temperature drop, and *A* is the surface area of interface.

#### Finite Element Models

COMSOL [36] Multiphysics 5.3a is used to simulate the thermal transport process in the structures. Two rectangles of 0.35 × 0.15 mm are set as controlled systems. To reveal the effect of continuous skeleton, two configurations with initial temperature of 293.15 K are built. The heat source temperature is 323.15 K at lower boundary, and free convection condition is set for upper boundary. To demonstrate the effect of nanofins, graphene skeleton with and without nanofins is constructed within 10 × 10 × 4 μm water cubes. Similarly, a high temperature of 323.15 K and free convection condition are set at left and right boundary, respectively, while the periodic boundary condition is fixed in the *z*-direction. The transient temperature difference is conducted within 8 × 10^–6^ s before the system approach to the stable equilibrium state. In this FEM calculation, interfacial thermal resistance *R*_*b*_ is obtained from MD simulations, while other parameters are empirical parameters (Table 2).

**Table 2:** The parameters used in finite element simulation by COMSOL

Parameters	*k*_graphene_ (W m^−1^ K^−1^)	*k*_water_ (W m^−1^ K^−1^)	*R*_*b*_ (K m^2^ W^−1^)	*T*_surrounding_ (K)
Value	250 [48]	0.6	6.7 × 10^–9^	293.15

## Results and Discussion

### Characterization of Graphene-Based Structures

We fabricate three types of samples, i.e., graphene nanoplates (GN), 3D graphene structure (3D-GS) and 3D graphene structure with covalent-bonding nanofins (3D-GS-CBF). GN is synthetized via the modified Hummer’s method, while 3D-GS and 3D-GS-CBF are prepared through plasma-enhanced chemical vapor deposition (PECVD). GN is dispersedly distributed without framework, exhibiting a petal-liked nanosheet morphology and soft-layered structure with the lateral size of several micrometers (Fig. S1). The discrete GN distribution in aqueous solutions is lack of continuous heat transfer pathways, resulting in strong phonon scattering between the graphene nanoplates (Fig. 1a). Unlike the GN, 3D-GS has a continuous skeleton structure with pores of which the size is ranging from dozens to hundreds of micrometers (Fig. 1c). Our high-magnification SEM images (Fig. 1d) suggest the skeleton surface of the 3D-GS is smooth composing of multilayer graphenes (Fig. 1e).

3D-GS-CBF shows a similar 3D porous skeleton structure with 3D-GS (Fig. 1c, f). Meanwhile, a large amount of graphene nanofins is distributed on the skeleton surface uniformly (Fig. 1f). Based on the cross-view SEM image (Fig. 1g), graphene nanofins with a typical height of ~ 400 nm and sharply exposed edges are arranged perpendicularly to the skeleton surface, which is able to utilize the extraordinarily high in-plane thermal conductivity of graphene. Moreover, Fig. 1h indicates the connections between nanofins and skeleton are covalent bonds, implying a very small thermal resistance at the interface for heat flowing from skeleton to nanofins effectively. At the same time, our Raman spectra results show the intensity ratio between D band and G band (*I*_D_/*I*_G_) is about ~ 1.21, also indicating that there are numerous graphene nanofins with edges in the 3D-GS-CBF (Fig. S2).

### Thermal Properties Measurements of the Graphene-Based Structures

**Fig. 2:**
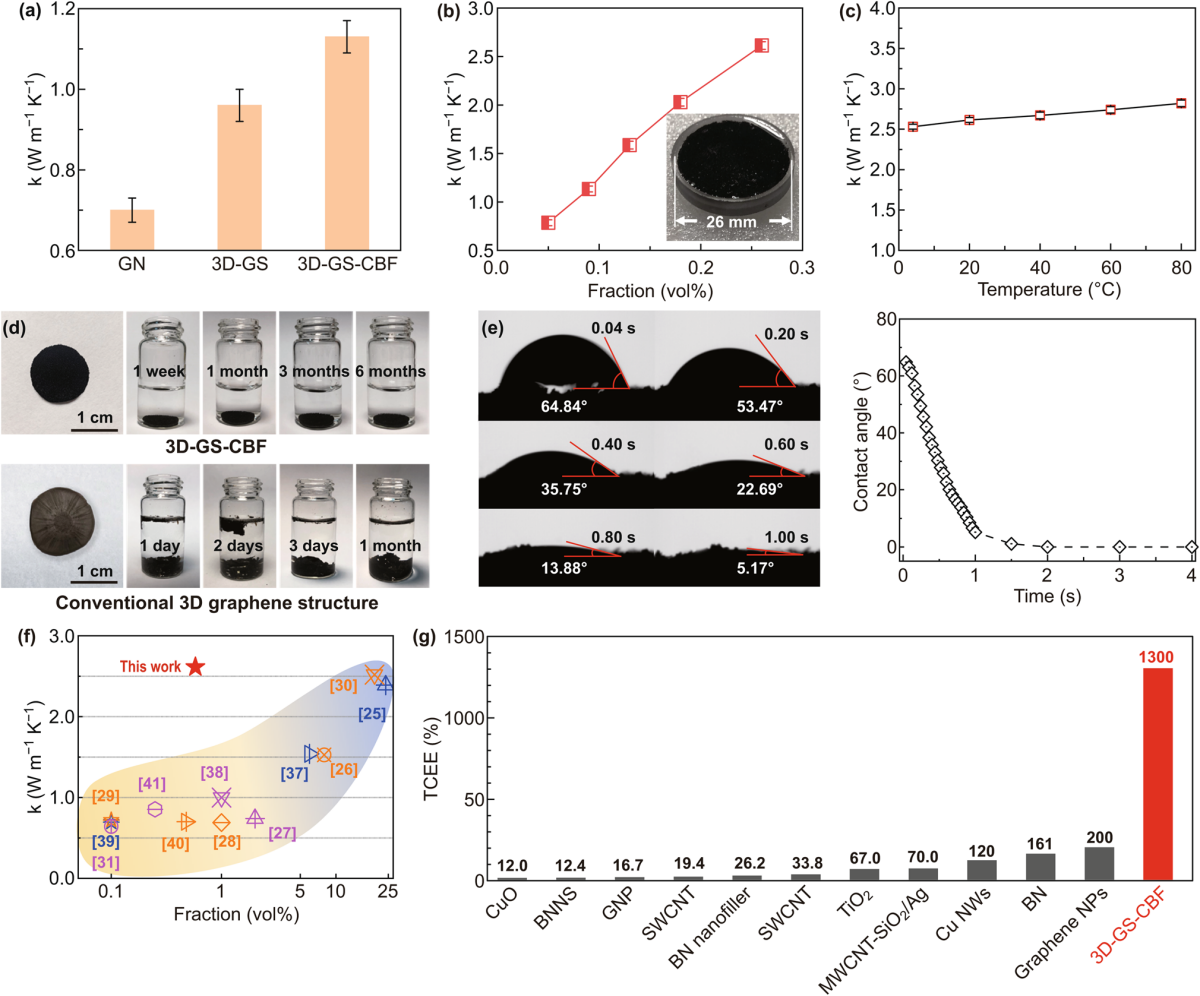
**a** Thermal conductivity k of GN, 3D-GS and 3D-GS-CBF aqueous mediums at the same volume ratio. **b** Thermal conductivity k of 3D-GS-CBF aqueous medium as a function of filler content. **c** Temperature dependence of thermal conductivity for 3D-GS-CBF aqueous medium. **d** Stability of 3D-GS-CBF (upper) and conventional graphene structures from chemical methods (down) in water. **e** Optical images of the apparent contact angle changes on 3D-GS-CBF surface. Comparison of **f** thermal conductivity and **g** TCEE between our work and prior studies (details can be found in Table S1)

Next, we suggest the GN, 3D-GS, and 3D-GS-CBF sample as the fillers for aqueous mediums and measure their thermal transport properties. The thermal conductivity *k* of GN aqueous medium is 0.66 W m^−1^ K^−1^ with the GN volume fraction of 0.1 vol% (Fig. 2a), of which the value is comparable to the existing results [28, 29, 31]. When 3D-GS is used as the filler in the aqueous medium, the thermal conductivity is 0.96 W m^−1^ K^−1^ at the same volume fraction, which is 45.5% higher than that of GN counterpart. *k* value of the aqueous medium can be further enhanced (by 71.2%) once the same volume fraction of 3D-GS-CBF is added into the water, demonstrating its potential as efficient heat dissipation materials. In addition, we also study the effect of volume fraction of the filler, i.e., 3D-GS-CBF. With improving the volume fraction of 3D-GS-CBF in the aqueous medium to 0.26 vol%, the apparent thermal conductivity *k* will increase to a record high value of 2.61 W m^−1^ K^−1^ with a thermal conductivity enhancement (TCE) of 355% (Fig. 2b).

We also characterize the long-term thermal and mechanical stability of 3D-GS-CBF sample in aqueous medium that is crucially important for practical applications. Due to the structure confinement of the 3D-GS-CBF which may restrict the movement of the water flow, its thermal conductivity shows a weak temperature dependence (Fig. 2c), e.g., 2.53 W m^−1^ K^−1^ (at 4 °C) and 2.82 W m^−1^ K^−1^ (at 80 °C), demonstrating the good thermal stability for practical long-term work. On the other hand, our 3D-GS-CBF sample shows that the structure can be stable in the solution even after 6 months, addressing the stability issues (e.g., folded graphene structure and sedimentation) of conventional non-covalent graphene networks as aqueous medium fillers (Fig. 2d), which can be interpreted by the robust 3D covalent structures and strong interfacial graphene–water interactions as discussed below. The optical microscope images of apparent contact angle suggest that water droplet will spread rapidly on the 3D-GS-CBF surface and the equilibrium contact angle is ~ 5.17° at 1 s (Fig. 2e), demonstrating its super-hydrophilic nature. The good surface wettability yields strong graphene–water interactions, thereby leading to lower interfacial thermal resistance. This can also be evidenced by the high-resolution X-ray photoelectron spectroscopy (XPS) spectra (Fig. S3). Oxygen-containing groups (e.g., C–OH, C=O, and O=C–H peaks) can be identified on the surface of 3D-GS-CBF, which is beneficial for enhancing the interfacial thermal transport.

To demonstrate the merits of our structure design, we also compare the thermal transport property of 3D-GS-CBF aqueous medium with other existing aqueous mediums with various fillers. Our aqueous medium exhibits an excellent thermal conductivity with only 0.26 vol% of 3D-GS-CBF, which is comparable to that of boron nitride (BN) nanosheets filling aqueous medium with over 20 vol% of BN fillers (Fig. 2f). As a result, the TCEE, i.e., the enhancement in heat conduction per 1% filler loading, of 3D-GS-CBF aqueous medium is as high as ~ 1300% and much larger than those of all other structure filling aqueous mediums (e.g., 200% of graphene nanoplates, 161% of BN, 120% of Cu nanowires and 67% of TiO_2_) [25–31, 37–41], which implies the superior heat transfer efficiency of our 3D-GS-CBF aqueous medium with respect to other systems (Fig. 2g).

### Multiscale Modeling the Thermal Transport Process in the Graphene-Based Structures

**Fig. 3:**
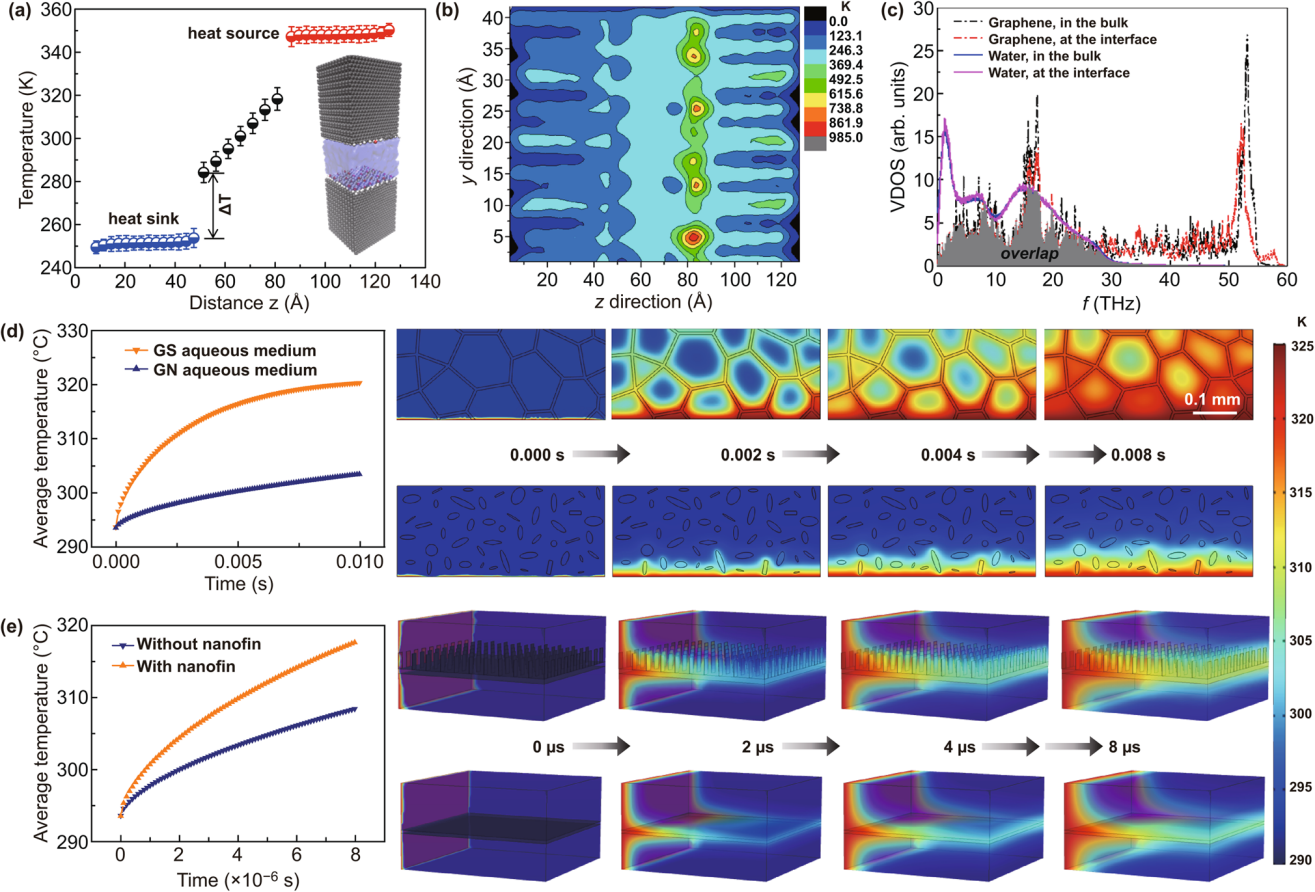
**a** 1D and **b** 2D temperature distribution at the graphene–water interface. **c** VDOS of graphene and water at the interface. **d** Averaged temperature of the GN and 3D-GS aqueous medium. **e** Average temperature of 3D-GS-CBF and 3D-GS aqueous mediums during the finite element method simulation

To investigate the underlying mechanisms of the thermal transport process, we implement the multiscale modeling approach, i.e., non-equilibrium molecular dynamics (NEMD) simulations and heat conduction equations, to capture the detailed microscopic information. The interfacial thermal conductance between graphene and water is firstly calculated via NEMD simulations and is then used in the heat conduction models. Based on the temperature distribution (Fig. 3a), the interfacial resistance between graphene and surrounding aqueous medium can be calculated (*R*_*b*_ = 6.7 × 10^–9^ K m^2^ W^−1^ [42, 43]), much lower than the reported values in previous work. The 2D temperature distributions (Fig. 3b) further confirm the efficient heat exchange at the graphene–water interface, which is probably because the exposed graphene edges can vibrate more frequently or easily due to the weak confinement. In order to gain more insights, we also study the similarity in the vibrational density of states (VDOS) by taking the Fourier transform of the velocity autocorrelation functions of atoms in an equilibrium state. As evidenced in Fig. 3c, a high overlap of vibration modes of graphene edges and adjacent water in the low-frequency region (< 30 THz) is observed, which can be interpreted by the strong interfacial interactions between graphene skeleton/nanofins/edges enriched with functional groups and surrounding water.

Based on the NEMD results, heat diffusion simulations are carried out to study the temperature evolution and distribution of the graphene structures. All the parameters used in the heat conduction equations are listed in Table 1 (Method). In the heat diffusion simulations, constant temperatures and free convection conditions are set for both the lower and upper boundaries. We find that the average temperature of GS aqueous medium increases more rapidly (by 16.9 K) than that of conventional GN aqueous medium (Fig. 3d), indicating the importance of continuous skeleton in improving the heat conduction. To study the nanofin effect on the heat conduction, we also study the thermal transport process in the 3D-GS-CBF aqueous medium (Fig. 3e). Our simulation results clearly demonstrate the merits of nanofin structures in enlarging the heat conduction at the graphene–water interfaces. Thereby, the multiscale simulation well validates the above experimental observations.

### Practical Thermal Applications

**Fig. 4:**
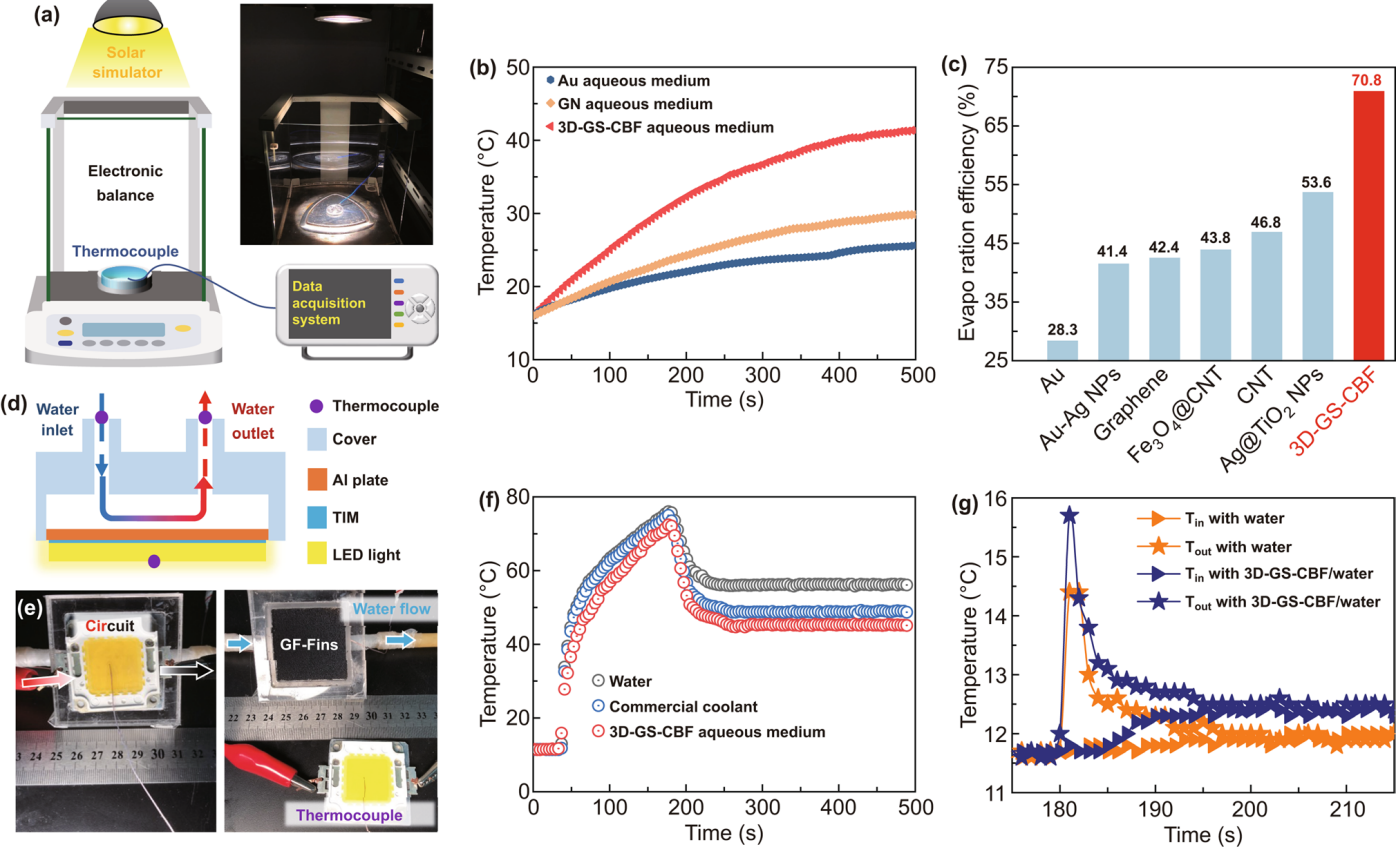
**a** Schematic of experimental setup for solar thermal conversion application. The apparatus includes a solar simulator, an electronic balance, thermocouples and data acquisition system. The inset denotes the photograph of apparatus. **b** Temperature of Au, GN and 3D-GS-CBF aqueous mediums as a function of illumination time at 1 sun. **c** Comparison of solar vapor generation efficiency between our work and previous studies. **d** Schematic of experimental setup for LED thermal management application. Water flows through the block from inlet to outlet. **e** Photographs of devices, showing the electric current and water flow. **f** Surface temperature of LEDs as a function of time for water, commercial coolant and 3D-GS-CBF aqueous mediums. **g** Water temperature at the inlet and outlet of LEDs as a function of time

Before closing, we apply our samples for two practical applications: solar thermal conversion and thermal management of LEDs. The experimental setup for solar thermal conversion application is presented in Fig. 4a. The main components of the setup include a solar simulator, container, data acquisition system, thermocouples and electric balance. Figure 4b shows the temperature rise of Au, GN and 3D-GS-CBF aqueous mediums. We find that the average temperature of 3D-GS-CBF aqueous mediums is much higher with respect to the other two situations. After 500 s illumination at 1 sun, the temperature of 3D-GS-CBF aqueous medium system increases by 25.4 °C, almost twofold higher than those of Au (9.6 °C) and GN counterparts (13.8 °C). As a result, the steady-state evaporation rate of 3D-GS-CBF reaches up to 1.13 kg m^−2^ h^−1^ (Fig. S4), while those of Au and GN cases are merely 0.50 and 0.74 kg m^−2^ h^−1^, respectively. As presented in Fig. 4c, we thus demonstrate that the solar vapor generation efficiency of 3D-GS-CBF aqueous medium (70.8%) is 2.5 and 1.7 times those of Au (28.3%) and GN (42.4%) counterparts, respectively, obviously superior to those of conventional aqueous mediums (e.g., 41.4% of Au–Ag [44], 43.8% of Fe_3_O_4_@CNT [45], 46.8% of CNT [46] and 53.6% of Ag@TiO_2_ [47]). More importantly, we would like to note that our structure design can remarkably improve the solar thermal conversion performance of the volumetric/bulk heating systems, which can be even comparable to those of interfacial solar evaporation system (i.e., evaporation rate of 1.0 ~ 1.3 kg m^−2^ h^−1^ and efficiency of 50~90%) with the optimized light adsorption capability (e.g., hierarchical architecture), rational thermal management (e.g., double-layer structure) to reduce heat loss and ingenious water pathway design, etc., paving a new way of enhancing the inherent heat transfer efficiency of aqueous mediums to achieve high-performance volumetric solar-driven vapor generation system.

The second practical application example is the thermal management of LEDs. Here, we study the cooling process of LEDs via pure water, 3D-GS-CBF and commercial coolant (i.e., water/ethylene glycol). The corresponding experimental setup is presented in Fig. 4d, e. LEDs are attached on the water-cooling apparatus through the thermal interfacial material layer. The heat generated by LEDs is dissipated by flowing water in the interspace of water block enclosed by the Al plate and PMMA cover. The surface temperature and operating current of the LEDs are measured by thermocouples and source meter, respectively. The surface temperature of LEDs rapidly increases to 157 °C within 146 s, and the operating current reaches up to 697.6 mA (Fig. S5), indicating the serious overheating phenomenon that will result in reduced working stability and service life. Applying the pure water to cool the system (at 176 s), the surface temperature of LEDs significantly decreases by 20 °C within 69 s. Importantly, the 3D-GS-CBF aqueous medium could have a better cooling effect, resulting in a lower working temperature of 45.2 °C (Fig. 4f), which is lower than those of commercial coolant (49.0 °C) and pure water system (56.1 °C). Moreover, 3D-GS-CBF aqueous medium is able to reduce the operation current by 18.3% (from 447.8 mA to 378.6 mA in 50 s) as shown in Fig. S6, which is quite good for reducing the energy cost. Meanwhile, the water temperature at the inlet and outlet of LEDs as a function of time is also measured to reveal the heat dissipation process (Fig. 4g). In the initial stage, the water temperature at the outlet increases largely because of the high temperature of LEDs and then gradually decreases to equilibrium state. Compared with pure water, 3D-GS-Fin aqueous medium can notably increase the outlet water temperature (by 4.0 °C), indicating an efficient heat exchange between water and LEDs.

## Conclusions

In conclusion, 3D graphene continuous structure with covalent-bonding nanofins is fabricated through PECVD method to realize high thermally conductive aqueous mediums. Our results show that 3D-GS-CBF can enhance the thermal transport properties of its corresponding aqueous medium by ~ 4.4-fold (2.61 W m^−1^ K^−1^) with an ultralow loading of 0.26 vol%, yielding a record high TCEE of 1300%, which is almost orders of magnitude larger than those in the state-of-the-art studies (< 200%). The multiscale simulations suggest that the large amount of nanofins on the top of graphene skeleton will increase the surface ratio largely and then increase heat exchange at the graphene–water interfaces significantly. As a result, in comparison with GN counterpart, the excellent thermal transport properties of 3D-GS-CBF aqueous medium can largely enhance the evaporation rate by 1.5-fold (up to 1.13 kg m^−2^ h^−1^) and its corresponding efficiency by 1.7-fold (up to 70.8%) in the solar thermal conversion applications. Moreover, the 3D-GS-CBF aqueous medium can also reduce the operation current by 18.3%, demonstrating a superior thermal management performance over commercial coolant. This work suggests a new venue of fabricating 3D continuous, covalent graphene networks to prepare high thermally conductive aqueous medium for high-performance advanced thermal applications. As a prime example, improving the thermal property can potentially increase the temperature rising rate of aqueous medium and thus boost the reaction kinetics of photocatalytic reactions, which deserves to be investigated in future work.

## Electronic supplementary material

Below is the link to the electronic supplementary material.

Supplementary file1 (PDF 320 kb)
