# Supplementary material for: Highly Thermo-Conductive Three-Dimensional Graphene Aqueous Medium
Source: Nanomicro Lett. 2020 Jul 1;12:138. doi: 10.1007/s40820-020-00478-2 (PMC7770698; doi:10.1007/s40820-020-00478-2)
Supplement: Supplementary file 1 — Supplementary file1 (PDF 320 kb) [file 40820_2020_478_MOESM1_ESM.pdf]

Supporting Information for

Highly Thermo-Conductive Three-Dimensional Graphene Aqueous Medium

Zheng Bo[1, 2], Chongyan Ying[1, 2], Huachao Yang[1, 2, *], Shenghao Wu[1, 2], Jinyuan Yang[1, 2], Jing Kong[1, 2], Shiling Yang[1, 2], Yanguang Zhou[3], Jianhua Yan[1], Kefa Cen[1]

[1]State Key Laboratory of Clean Energy Utilization, Institute for Thermal Power Engineering, College of Energy Engineering, Zhejiang University, 38 Zheda Road, Hangzhou 310027, People's Republic of China

[2]ZJU-Hangzhou Global Scientific and Technological Innovation Center, Hangzhou 311215, People's Republic of China

[3]Department of Mechanical and Aerospace Engineering, The Hong Kong University of Science and Technology, Clear Water Bay, Kowloon, Hong Kong, China

*Corresponding author. E-mail: huachao@zju.edu.cn (Huachao Yang)

Supplementary Figures and Table

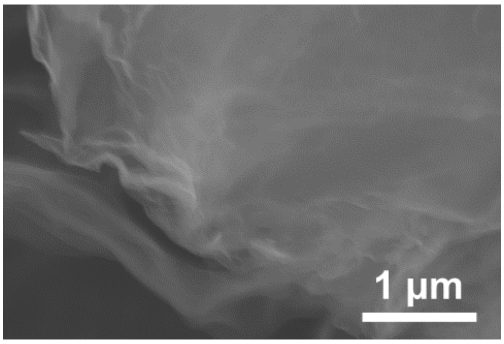

Fig. S1 SEM image of petal-like structure of GN

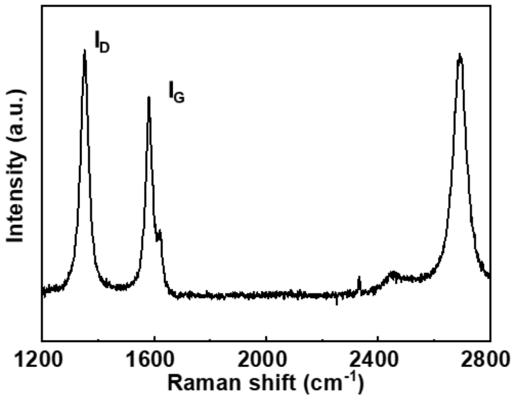

Fig. S2 Raman spectra of 3D-GS-CBF

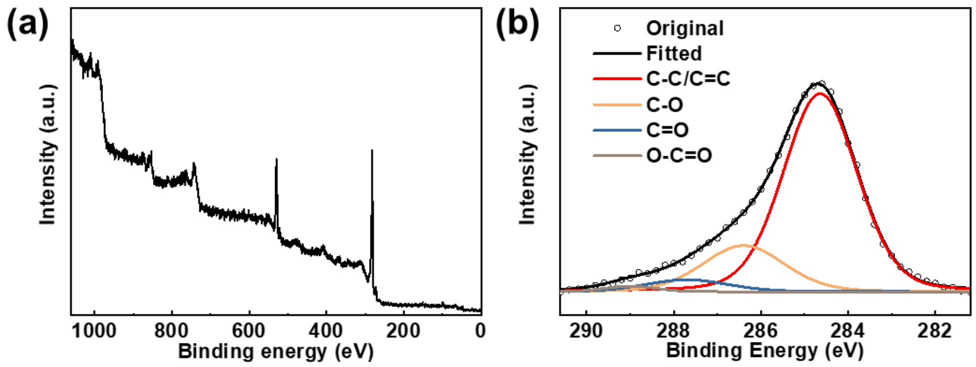

Fig. S3 a XPS survey spectra and **b** Gaussian line fitted C 1s spectra of 3D-GS-CBF

Table S1 Comparison of thermal conductivity of our work with previous studies

Filler	Fraction	*k* (W m^{-1} K^{-1})	TCE (%)	TCEE (%)	References
BNNS	24 vol%	2.39	298	12.4	2018 [S1]
GNP	0.1 vol%	0.69	16.7	16.7	2018 [S2]
SWCNT	0.48 vol%	0.7	16.2	33.8	2016 [S3]
CuO	2 vol%	0.74	24	12	2016 [S4]
TiO_2	1 vol%	1.0	67	67	2013 [S5]
BN	0.1 vol%	0.696	16.08	160.8	2016 [S6]
Cu nanowires	0.25 vol%	0.854	40	120	2017 [S7]
graphene nanoplatelets	0.44 vol%	0.72	20	200	2015 [S8]
BN nanofiller	6 vol%	1.54	157	26.2	2011 [S9]
MWCNT-SiO_2/Ag	0.1 vol%	0.642	7	70	2017 [S10]
FGNs	20 vol%	2.52	320	16	2019 [S11]
SWCNT	8 vol%	1.528	155	19.4	2017 [S12]
**3D-GS-CBF**	**0.26 vol%**	**2.61**	**335**	**1300**	**This work**

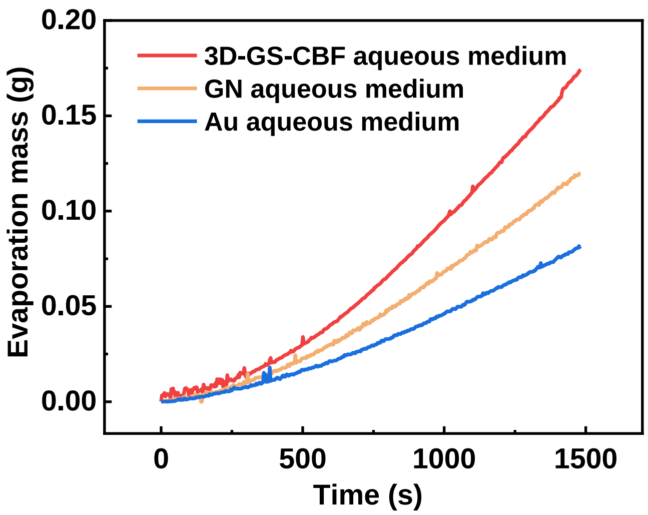

Fig. S4 Evaporation amount of different systems under 1 sun light intensity

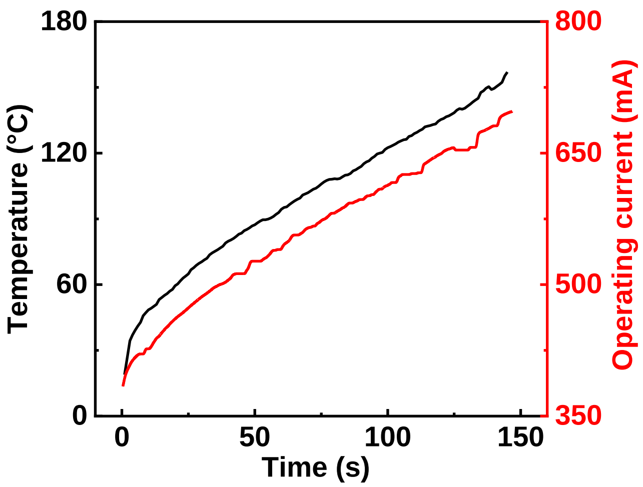

Fig. S5 Surface temperature and operating current of LEDs without thermal management

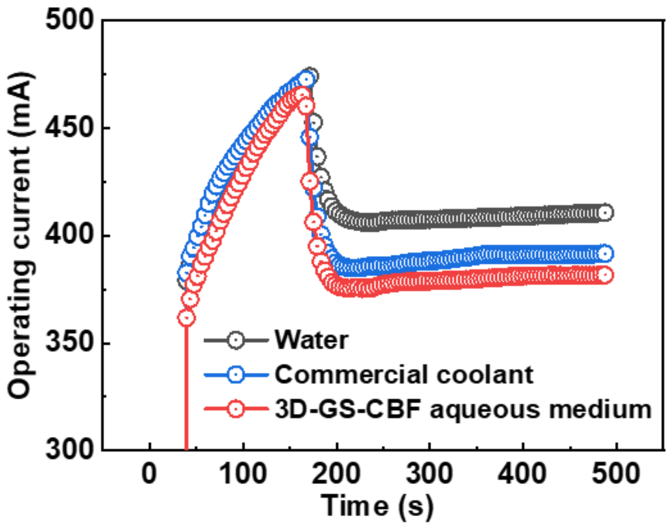

Fig. S6 Operating current of LEDs as a function of time under thermal management of water, commercial coolant and 3D-GS-CBF aqueous mediums

Supplementary References

[S1] Hou X, Wang M, Fu L, Chen Y, Jiang N, Lin CT, Wang Z, Yu J, Boron nitride nanosheet nanofluids for enhanced thermal conductivity. Nanoscale. **10** (27), 13004-13010 (2018). https://doi.org/10.1039/c8nr00651b

[S2] Wang Y, Al-Saaidi HAI, Kong M, Alvarado JL, Thermophysical performance of graphene based aqueous nanofluids. Int. J. Heat Mass Transf. **119** 408-417 (2018). https://doi.org/10.1016/j.ijheatmasstransfer.2017.11.019

[S3] Xing M, Yu J, Wang R, Experimental investigation and modelling on the thermal conductivity of cnts based nanofluids. Int. J. Therm. Sci. **104** 404-411 (2016). https://doi.org/10.1016/j.ijthermalsci.2016.01.024

[S4] Agarwal R, Verma K, Agrawal NK, Duchaniya RK, Singh R, Synthesis, characterization, thermal conductivity and sensitivity of cuo nanofluids. Appl. Therm. Eng. **102** 1024-1036 (2016). https://doi.org/10.1016/j.applthermaleng.2016.04.051\

[S5] Reddy MCS, Rao VV, Experimental studies on thermal conductivity of blends of ethylene glycol-water-based tio2 nanofluids. Int. Commun. Heat Mass Transf. **46** 31-36 (2013). https://doi.org/10.1016/j.icheatmasstransfer.2013.05.009

[S6] Krishnam M, Bose S, Das C, Boron nitride (BN) nanofluids as cooling agent in thermal management system (tms). Appl. Therm. Eng. **106** 951-958 (2016). https://doi.org/10.1016/j.applthermaleng.2016.06.099

[S7] Bhanushali S, Jason NN, Ghosh P, Ganesh A, Simon GP, Cheng W, Enhanced thermal conductivity of copper nanofluids: The effect of filler geometry. ACS Appl. Mater. Interfaces. **9** (22), 18925-18935 (2017). https://doi.org/10.1021/acsami.7b03339

[S8] Sadeghinezhad E, Togun H, Mehrali M, Sadeghi Nejad P, Tahan Latibari S, Abdulrazzaq T, Kazi SN, Metselaar HSC, An experimental and numerical investigation of heat transfer enhancement for graphene nanoplatelets nanofluids in turbulent flow conditions. Int. J. Heat Mass Transf. **81** 41-51 (2015). https://doi.org/10.1016/j.ijheatmasstransfer.2014.10.006

[S9] Zhi C, Xu Y, Bando Y, Golberg D, Highly thermo-conductive fluid with boron nitride nanofillers. ACS Nano **5** (8), 6571-6577 (2011). https://doi.org/10.1021/nn201946x

[S10] Zeng J, Xuan Y, Enhanced solar thermal conversion and thermal conduction of mwcnt-sio2/ag binary nanofluids. Appl. Energy **212** 809-819 (2018). https://doi.org/10.1016/j.apenergy.2017.12.083

[S11] Zhao H-R, Ding J-H, Ji D, Xu B-Y, Yu H-B, Highly thermoconductive fluid with aqueous compatible graphene. Mater. Res. Express. **6** (5), (2019). https://doi.org/10.1088/2053-1591/ab019e

[S12] Arani AAA, Akbari OA, Safaei MR, Marzban A, Alrashed AAAA, Ahmadi GR, Nguyen TK, Heat transfer improvement of water/single-wall carbon nanotubes (swcnt) nanofluid in a novel design of a truncated double-layered microchannel heat sink. Int. J. Heat Mass Transf. **113** 780-795 (2017). https://doi.org/10.1016/j.ijheatmasstransfer.2017.05.089
